# Genetics of Childhood Steroid Sensitive Nephrotic Syndrome: An Update

**DOI:** 10.3389/fped.2019.00008

**Published:** 2019-01-29

**Authors:** Brandon M. Lane, Rachel Cason, Christopher Imokhuede Esezobor, Rasheed A. Gbadegesin

**Affiliations:** ^1^Division of Nephrology, Departments of Pediatrics, Duke University Medical Center, Durham, NC, United States; ^2^Duke Molecular Physiology Institute, Duke University Medical Center, Durham, NC, United States; ^3^Department of Pediatrics, College of Medicine of the University of Lagos, Lagos, Nigeria

**Keywords:** nephrotic syndrome, SSNS, podocyte, MHC class II LOCUS, HLA DQ/DR

## Abstract

Advances in genome science in the last 20 years have led to the discovery of over 50 single gene causes and genetic risk loci for steroid resistant nephrotic syndrome (SRNS). Despite these advances, the genetic architecture of childhood steroid sensitive nephrotic syndrome (SSNS) remains poorly understood due in large part to the varying clinical course of SSNS over time. Recent exome and genome wide association studies from well-defined cohorts of children with SSNS identified variants in multiple MHC class II molecules such as HLA-DQA1 and HLA-DQB1 as risk factors for SSNS, thus stressing the central role of adaptive immunity in the pathogenesis of SSNS. However, evidence suggests that unknown second hit risk loci outside of the MHC locus and environmental factors also make significant contributions to disease. In this review, we examine what is currently known about the genetics of SSNS, the implications of recent findings on our understanding of pathogenesis of SSNS, and how we can utilize these results and findings from future studies to improve the management of children with nephrotic syndrome.

## Childhood Nephrotic Syndrome Overview

Nephrotic syndrome (NS) is the most common glomerular disease seen in the pediatric age group. It is the second most common kidney disease seen in pediatric nephrology clinic, the most common being congenital malformations of the kidney and the urinary tract. It is characterized by massive proteinuria, hypoalbuminemia, peripheral edema and hyperlipidemia (1). The prevalence and incidence of NS is unknown, however estimates from different studies suggest that its incidence may be about 1.5 to 16.9 cases per 100,000 children and prevalence is about 16 cases per 100,000 children. Its incidence and prevalence varies between different geographical regions of the world and ethnicities (2). In a recent study in Ontario, Canada, Banh et al. reported incidence rate of 2.40, 15.83, 1.81, 3.01 for children of European, South Asian, East/Southeast Asian, and African ancestries, respectively (3).

Nephrotic syndrome in children is generally classified into steroid sensitive nephrotic syndrome (SSNS) and steroid resistant nephrotic syndrome (SRNS) based on the initial response to corticosteroid therapy at presentation. This classification resulted from the International Study of Kidney Disease in Children (ISKDC) studies in the 1970s, which showed that irrespective of morphological changes on kidney biopsy, the most important prognostic factor in NS is the response to corticosteroid and other immunosuppressive therapies (4). Children with SSNS have excellent prognosis with >95% unlikely to progress to end stage kidney disease (ESKD) as long as they remain therapy responsive. On the other hand, the majority of children with SRNS will have rapid progression of their disease and will be in ESKD within 5 years of diagnosis (5). Just as differences exist in the prevalence and incidence of NS in different populations, there are also differences in pattern of response to therapy; SSNS is more common in Asian children compared with other ethnicities, and children of Hispanic and African descents are more likely to have SRNS type of NS, suggesting that genetic factors may play a role in both the incidence of NS and pattern of response to therapy (3).

While the most important classification of NS is based on initial response to therapy, other classification schemas exist as well. For example, children with onset of nephrotic syndrome in the first 3 months of life are classified as having congenital nephrotic syndrome (CNS) and it is well known that about 80% of children with CNS will have genetic NS, with most being therapy resistant (6). While outcomes are heavily dependent on pattern of therapy response, morphologic findings from kidney biopsy also has prognostic significance. For example, more than 80% of children with minimal change disease (MCD) will have therapy responsive disease and therefore excellent prognosis. On the other hand, most children with focal and segmental glomerulosclerosis (FSGS) will have therapy resistant disease that is characterized by rapid disease progression to ESKD within 5–10 years of diagnosis (7, 8).

The etiology of idiopathic childhood nephrotic syndrome has remained elusive for decades. However, secondary causes such as infections (hepatitis B & C, HIV, etc.), malignancy (leukemia, lymphoma, other T cell associated cancers), autoimmune disorders (systemic lupus erythematosus, IgA nephropathy, and other immune complex mediated disorders, etc.), and drug associated (non-steroidal anti-inflammatory drugs, certain antibiotics) have been reported (1). More recently advances in genomics and molecular biology have led to identification of over 50 single gene causes of SRNS as well as several genetic risk factors for different morphologic forms of SRNS. However, the contribution of genetic factors to the prevalence and clinical course of SSNS have been harder to define, likely due to a more complex pattern of inheritance and variable phenotypic expression that is characterized by relapse and remission. In this review, we will discuss what is currently known about the genetics of SSNS, provide evidence that there are important genetic drivers of SSNS in different populations, and discuss future approaches to defining the genetic architecture of SSNS.

### Pathogenesis

Massive urinary loss of proteins the size of albumin or greater is the hallmark of nephrotic syndrome (2). Other features of NS include generalized edema, hyperlipidemia, and hypoalbuminemia. In addition, patients with NS are at an increased risk of acute kidney injury, thromboembolic events and infections (9–12).

#### Proteinuria

The glomerular filtration barrier (GFB) effectively limits the urine loss of albumin and other higher molecular weight proteins to <100 mg/m^2^ per 24 h. This barrier consists of three distinct layers, namely the fenestrated endothelial cells, the glomerular basement membrane, and the specialized visceral epithelial cells, the podocytes. The foot processes of adjacent podocytes interdigitate to form the slit diaphragm. Our recent understanding of the glomerular function now credits the slit diaphragm as a major barrier to the filtration of albumin (13). However, factors responsible for the disruption of the GFB are not completely known and are subject of ongoing investigations.

#### Role of T and B Cells in the Pathogenesis of Nephrotic Syndrome

Accumulated evidence points to the role of T and B cell disorders in the pathogenesis of steroid sensitive nephrotic syndrome (SSNS). All current therapeutic agents for SSNS act by either depleting T and B cell populations or by modulating their functions (14). For example, in children with SSNS characterized by steroid dependent or frequently relapsing course, maintenance of remission is commonly achieved with calcineurin inhibitors and anti-proliferative agents such as mycophenolate mofetil, both agents are known to maintain remission through their effects on T cell function.(15, 16). In addition, NS may also present as paraneoplastic syndrome in some lymphoreticular malignancies. Furthermore, children with NS have been reported to achieve remission following measles infection, a disease that is known to suppress cell-mediated immunity (17, 18). Sustained remission of NS following administration of rituximab or ofatumumab, anti-CD20 monoclonal antibodies that deplete B cell population, and the occurrence of NS in some people with light chain gammopathy highlight the additional role of B cells in the pathogenesis of SSNS (19–21). To date, one of the strongest evidence for the role of adaptive immunity in the pathogenesis of SSNS is the association between SSNS and variants in the gene loci for the human leucocyte class II antigens (22–28). These genes are responsible for the production of surface proteins on immune cells required for antigen presentation.

The exact mechanism of how disorders in the immune systems cause increased permeability of the glomerular filtration barrier is not fully understood. However, dysregulation of different cytokines expressed by activated immune cells like the T cells have been reported during relapse of NS (29). Also, radical oxygen species are known to cause proteinuria in rats, and activated T cells cause imbalance in reactive oxygen species (30). Furthermore, some soluble permeability factor such as cardiotrophin-like cytokine 1 factor have been recovered from activated T cells (31). These circulating permeability factors are thought to alter podocyte structure and functions leading to disruption of the GFB and subsequently proteinuria.

#### Soluble Circulating Permeability Factors

Another theory that explains proteinuria in NS is the presence of soluble circulating permeability factors capable of increasing the loss of albumin and higher molecular weight proteins into the urine. This theory considers the normal kidney as being in an abnormal milieu (32). Several reported cases in the literature provide support for this theory. Notable among these are recurrence of NS following kidney transplantation in patients with NS especially those with primary focal segmental glomerulosclerosis (33, 34). Prevention or remission of proteinuria in these situations following plasmapheresis provides additional support for the role of circulating permeability factors. (35, 36) Transient neonatal proteinuria in children born to mothers with NS further strengthens the evidence for the presence of some circulating permeability factors (37). These putative circulating factors are thought to be in the 30–50 kDa range (38). Their strong affinity to galactose provides the theoretical basis for reports of some efficacy of galactose in the treatment of NS (39).

Some of the putative soluble circulating permeability factors include cardiotrophin-like cytokine 1 factor, soluble urokinase-like plasminogen receptor (suPAR), hemopexin, radical oxygen species, vascular endothelial growth factors, IL-13, IL-18, tumor necrosis factor-α, and angiopoetin-like 4 factor (40–42).

These circulating factors, likely acting together, exert their effects on the glomerular filtration barrier to cause proteinuria. For example, hemopexin reduces glomerular sialoproteins and alters the actin cytoskeleton, a major contributor to podocyte structural integrity (43, 44).

#### Podocytes

The discovery of over 50 single genes causing mostly SRNS highlights the primacy of the role of the podocytes in the pathogenesis of NS. Furthermore, current therapies of NS are known to have non-immune mediated effects on the podocyte, further underscoring its role in the pathogenesis of proteinuria in NS (45–48). The podocyte and slit diaphragm present a major barrier to protein loss in urine. Disturbance in podocyte structure or function is now regarded as the hallmark of NS (49). Hence the recognition of NS as a form of podocytopathy. Although, podocyte dysfunction or alteration in podocyte structure may be a primary event leading to proteinuria, circulating permeability factors, and disorders in T and B cell functions are thought to act via perturbation of podocyte function, structure or both (50).

#### Edema

Generalized edema is the most common presenting feature of NS. The exact mechanism is not known. Two theories provide explanation for the pathogenesis of edema in NS (51). The most compelling theory is the underfill theory which attributes the edema to the loss of oncotic pressure due to hypoalbuminaemia. The reduction in capillary oncotic pressure leaves the capillary hydrostatic pressure unchallenged, resulting in the net translocation of fluid to the interstitial space. The resulting drop in renal blood flow activates renal angiotensin system and avid conservation of salt and water in the kidney. Support for this theory includes the contracted intravascular volume and high serum renin and aldosterone levels seen in some children with NS (52, 53). Improvement in the edema with albumin infusion further supports the underfill theory. In contrast, not every child with NS has contracted intravascular volume and elevated renin or aldosterone level (53). Furthermore, the prompt disappearance of edema following response to corticosteroids or other immunosuppressants, even with low serum albumin further negates the primacy of the underfill theory. The overfill theory suggests that the cause of edema in NS is a primary increase in the reabsorption of sodium and water in the distal convoluted tubules (54). As suggested by Eddy and Symons, it is likely that the two theories may be at work in the same child to varying degree at different times (2).

#### Hyperlipidemia

Hypertriglyceridemia and hypercholesterolemia characterize most cases of NS. The dyslipidemia is due to a combination of increased liver synthesis of albumin and lipoprotein to compensate for urinary loss of albumin, and disturbance in the metabolism of cholesterol (55). Specifically, there is an uptick in the activity of 3-hydroxy-3-methylglutaryl-CoA reductase, involved in the synthesis of cholesterol, and a decrease in activity of enzymes such as lipoprotein lipase, and hepatic lipase, involved in the degradation of lipids (56). Corticosteroids and dietary changes during relapse of NS further contribute to the dyslipidemia associated with NS.

## Evidence That SSNS has Strong Genetic Component

As mentioned previously, unlike SRNS where over 50 monogenic causes have been identified, single gene causes of SSNS have remained relatively elusive. Nonetheless, several reports point to importance of genetic factors in the risk and pathogenesis of SSNS. First, about 3% of children with SSNS have a first degree relative with SSNS (57). Second, SSNS is more common in South Asians than Caucasians and other races in both Europe and North America (1, 3, 58). Additionally, risk variants for SSNS in the HLA-class I and II gene loci have been documented in Asian, European, Caucasian, and African-American children (22–28, 59). An important first step in adaptive immunity is the presentation of foreign antigens by antigen presenting cells to T cells. Once activated, the T cells initiate a cascade of immune reactions that characterize adaptive immunity. Human leucocyte antigens play an important role by acting as receptors for foreign antigens on the surfaces of antigen presenting cells.

### Recent Findings From Genetic Studies

#### Mendelian Inheritance

**Figure 1:**
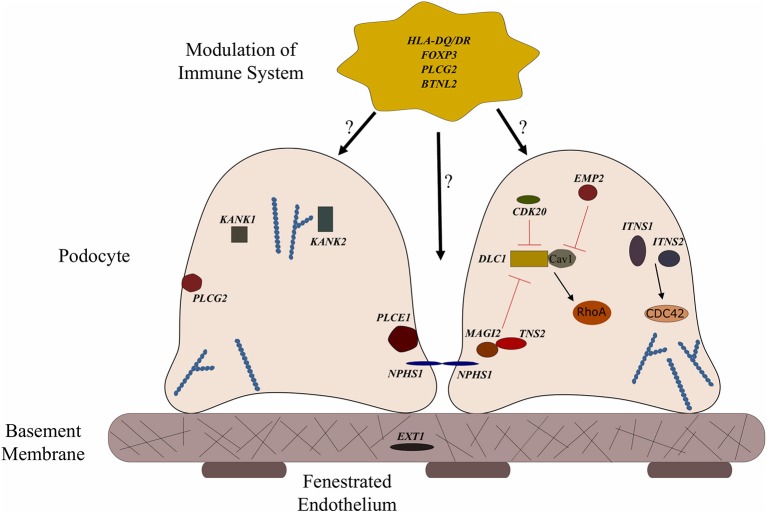
Genes associated with steroid sensitive nephrotic syndrome (SSNS). In the relatively rare cases of monogenic SSNS, the vast majority of genes that have been implicated in SSNS pathology locate to the glomerular filtration barrier and more specifically to the podocytes and slit diaphragm (Table 1). Among them are a key slit diaphragm protein Nephrin encoded by *NPHS1* which is known to interact with *MAGI2* and *TNS2*. These two proteins also interact as binding partners and along with *CDK20* act to influence podocyte cytoskeletal activity through negative regulation of *DLC1* activity (Red lines). *DLC1* encodes s a RhoGTPase activating protein for RhoA and its activity can be modulated by the product of *EMP2* through inhibition of the DLC1 binding partner Caveolin1 (CAV1). Intersectins 1 and 2 encoded by *ITSN1* and *ITSN2* act as GEFs for another small RhoGTPase, CDC42. The molecules encoded by *KANK1* and *KANK2* are also involved in RhoGTPase regulation and are known to affect actin polymerization. *PLCE1* and *PLCG2* encode proteins that modulate actin cytoskeleton and signaling at the slit diaphragm through calcium regulation. In addition to podocyte expression, *PLCG2* is expressed in lymphocytes and it is known to affect immune response signaling. Evidence suggests that most cases of SSNS involve modulation of the immune system, though it is not clear exactly how this immune dysregulation affects the integrity of the glomerular filtration barrier. MHC Class II variants in the *HLA-DR/DQ* region are likely key components of this altered immune response. Other candidate loci include another MHC class II associated molecule *BTNL2*, as well as a regulator of T cell activity encoded by *FOXP3. EXT1* is expressed in the glomerular basement membrane where it is involved in heparan sulfate biosynthesis.

To date, no single gene has been confirmed to cause SSNS exclusively, however, some children with mutations in SRNS genes such as *PLCE1* and *NPHS1* have been reported to have steroid responsive disease (Figure 1 and Table 1) (6, 64, 65). In addition, there are reports of children with NS and mutations in single genes *KANK 1 or 2, EXT 1* or *FOXP3* who responded to corticosteroids or have minimal change histology (Figure 1 and Table 1) (60–63). A recent study reported a Turkish family with SSNS and loss of function mutation in epithelial membrane protein 2 (*EMP2*) gene (Figure 1 and Table 1) (60). However, further analysis of a larger cohort of NS patients identified additional patients with both SSNS and SRNS carrying bi-allelic mutations in *EMP2*, suggesting that mutations in this gene may cause variable disease phenotype. Furthermore, analysis of a large cohort of 131 patients with familial SSNS (59 unrelated families) did not identify pathogenic variants in *EMP2* (59).

**Table 1:** Monogenic causes of SSNS.

**Gene**	**Locus**	**Type of mutation**	**Protein localization**	**Associated with SRNS**	**Extra renal manifestations**	**References**
*EMP2*	16p13	Missense, Truncating	Podocyte	Y	No	(60)
*EXT1*	8q23	Missense	Glomerular basement membrane	N	Multiple exostoses	(61)
*FOXP3*	Xp11	Missense	Immune cells	Y	Immunodeficiency, Polyendocrinopathy, Enteropathy	(62)
*KANK1*	9p24	Missense	Podocyte	Y	No	(63)
*KANK2*	19p13	Missense	Podocyte	Y	No	(63)
*NPHS1*	19q12	Missense	Podocyte and slit diaphragm	Y	No	(64), (65)
*PLCE1*	10q23	Truncating	Podocyte and slit diaphragm	Y	No	(66)
*MAGI2*	7q21	Truncating	Podocyte	Y	Neurologic impairment	(67)
*TNS2*	12q13	Missense	Podocyte	Y	Asthma, Hypertension, Short Stature	(67)
*DLC1*	8p22	Missense, Truncating	Podocyte	Y	Hypertension, Seizures, Visual Disturbance	(67)
*CDK20*	9q22	Missense	Podocyte	N	No	(67)
*ITSN1*	21q22	Missense	Podocyte	Y	No	(67)
*ITSN2*	2p23	Missense	Podocyte	N	No	(67)

More recently, a study focused on 17 families with partially treatment sensitive nephrotic syndrome (pTSNS), which includes patients with SSNS, as well as patients with partial response to corticosteroid treatment. Through a combination of homozygosity mapping (HM) and whole exome sequencing (WES) in these families as well as high throughput sequencing of an additional 1000 patients with NS, they identified recessive mutations in six genes (*MAGI2, TNS2, DLC1, CDK20, ITSN1*, and *ITSN2*) as novel causes of disease (Figure 1 and Table 1) (67). Additionally, this study provided evidence that these six genes act as part of a common Rho GTPase regulatory pathway in podocytes that when disrupted can lead to NS.

In a screen of 400 patients with NS however, Ashraf et al. identified homozygous truncating mutations in MAGI2 as a cause of disease in two patients with SRNS. This has been further confirmed in several additional patients with SRNS in an independent cohort (68). Membrane Associated Guanylate Kinase Inverted 2 (MAGI2) is a scaffolding protein that regulates podocyte cytoskeletal and slit diaphragm dynamics through interactions with proteins such as nephrin (Figure 1) (68, 69). *TNS2* encodes tensin2, a focal adhesion molecule that like *MAGI2* is known to regulate protein kinase B (PKB/AKT) activity and is vital to proper podocyte cytoskeletal dynamics(Figure 1) (70, 71). Whole exome sequencing of a patient with MCD uncovered a homozygous mutations in *TNS2* and sequencing of additional patients led to the discovery of five additional homozygous or compound heterozygous pathogenic mutations. WES of a patient diagnosed with membranoproliferative glomerulonephritis (MPGN) identified homozygous missense mutations in a highly conserved amino acid residue of cyclin dependent kinase 20 (*CDK20*). Ashraf et al. posit that the products of *MAGI2* and *TNS2* interact and that all three proteins affect RhoGAP activity in podocytes through interactions with, or modulations of DLC1 Rho GTPase-activating protein (Figure 1).

*In vitro* analysis by Ashraf et al. determined that the Rho GTPase-activating protein encoded by *DLC1* regulates RhoA activity, which is essential for maintaining podocyte cytoskeleton dynamics (Figure 1). High throughput sequencing of the NS cohort revealed four families with recessive mutations in *DLC1*. Further investigation of additional Rho GTPase modulators revealed mutations in Intersectin1 (*ITSN1*) and Intersectin2 (*ITSN2*) as the cause of early onset NS in several families. This included homozygous missense *ITSN1* mutations in two siblings in an Arab family and four other compound mutations in two additional families. WES of *ITSN2* in five members of a Japanese family with two affected individuals revealed compound heterozygous mutations as a cause of disease which was then confirmed through the discovery of homozygous missense mutations in an additional NS patient. Intersectins are a family of proteins involved in clatherin mediated endocytosis and Ashraf et al. demonstrate that the products of *ITSN1* and *ITSN2* act as GEFs regulating Cdc42 activity (Figure 1). Dysregulation of these small Rho GTPases has been shown to affect podocyte cytoskeleton dynamics leading to loss of GFB integrity in mice (72–74). The cytoskeletal effects of variants of this pathway are illustrated by reduced cell migration with knockdown of *MAGI2* and *CDK20*, and a loss of podocyte restoration after LPS induced podocyte foot process effacement in *ITSN2* homozygous mice. EMP2 is theorized to contribute to this pathway as well through its negative regulation of caveolin-1 expression, whose dysregulation is associated with NS (Figure 1). The interaction with and potential regulation of *DLC1* by caveolin-1 is lost in SSNS associated *DLC1* variants. Dexamethasone is hypothesized to contribute to disease remission by affecting this EMP2-DLC1 pathway and/or by directly affecting Rho GTPase activity. While the results of these studies are extremely valuable to our understanding of NS pathology, the variable steroid response in these patients and a lack of clear connections to the established role of immune dysregulation in SSNS suggests that additional factors may be involved. The genes that have been reported as possible monogenic causes of SSNS are listed in Table 1.

#### Complex Inheritance

Though several possible monogenic causes of SSNS have now been reported, Mendelian SSNS is still extremely rare. In addition, these genes are thought to exert their influence on disease primarily through their effects on podocytes. These proposed pathogenic mechanisms fail to explain the apparent contribution of immune dysregulation to SSNS. While corticosteroids and other immunosuppressive therapies may act through podocyte cytoskeletal effects, such as those outlined by Ashraf et al. there have been several SSNS risk loci identified that suggest a more complex immune mediated mechanisms (Table 2). The majority of identified SSNS risk loci are located in the *HLA-DQ* and *HLA-DR* region of the human leukocyte antigen (HLA) gene complex encoding MHC class II molecules required for extracellular antigen presentation (24, 25, 75–79). These *HLA-DQ* and *HLA-DR* regions are highly polymorphic, a characteristic that is essential for creating a robust adaptive immune system, however, specific variants have been linked to the development of autoimmune diseases.

**Table 2:** SSNS studies implicating MHC II loci.

**Study population**	**Cohort size (*n*)**	**Gene**	**References**
UK Caucasian	40	*HLA-DR7*	(75)
		*HLA-DQW2*	
U.S. Caucasian	32	*HLA-DQW2*	(76)
French and German	161	*HLA-DQB*	(24)
		*HLA-DQA*	
Japanese	24	*HLA-DQB1*	(25)
Japanese	30	*HLA-DQA1*	(77)
		*HLA-DQB1*	
Chinese (Taiwan)	59	*HLA-DQB1*	(78)
		*HLA-DR*	
French	75	*HLA-DQA1*	(79)
Dutch	146		
British	335		
South Indian	76	*HLA DRB1*	(23)
		*HLA DQB1*	
South Asian	214	*HLA-DQA1*	(22)
U.S. white	100		
European	323	*HLA-DQA1*	(80)
African American	65	*HLA-DQA1*	(26)
European, North African, Southeast Asian	131	*HLA-DQA1*	(59)
Japanese	224	*HLA-DR*	(28)
Japanese	216	*HLA-DQ*	
French caucasian	132	*HLA-DR*	(27)
European caucasian	133	*HLA-DQ*	
African	56		
Maghrebian	85		

Of these *HLA* genes, variants in the *HLA-DR* locus were the first to be associated with NS, with variants being linked to disease in a cohort of Japanese children with SSNS (25). However, due to the strong LD between the *DR* and *DQ* regions, the specific contributions of the *DR* and *DQ* have only recently been examined. In an unbiased exome wide analysis, Gbadegesin et al. identified 4 variants in the *HLA-DQA1/HLA-DQB1* locus associated with SSNS in a South Asian cohort (22). The two *HLA-DQA1* variants, rs1129740 and rs1071630, were then replicated in an independent European SSNS cohort. The follow up study revealed that these variants are also associated with SSNS in African American children (26). This locus has now been confirmed in several other independent studies (Table 3) (27, 28, 80). *HLA* imputation in the same cohort of South Asian children revealed HLA-DRB1^*^07:01, HLA-DQA1^*^02:01, HLA-DQB1^*^02:01, and HLA-DQA1^*^01:01 as the SSNS associated classic *HLA* alleles (26). These findings seem to suggest that the *HLA-DQA1* locus is a cosmopolitan risk allele and it is unlikely to be the explanation for difference in prevalence and severity of SSNS seen in different ethnicities. In depth analysis of *HLA-DQA1* at the amino acid level by Adeyemo et al identified strong associations with variations at positions, 76, 56, 69, and −16 (26). The functional implications of these amino acid positions are still unclear but positions 76 and 56 appear to be involved in peptide binding and display potential associations with the development of gluten sensitivity suggesting a possible link between food allergy and SSNS.

**Table 3:** *HLA* SNPs and classic alleles identified in SSNS cohorts.

**SNP**	**Adeyemo et al. (26)**	**Jia et al. (28)**	**Debiec et al. (27)**	**Sekula et al. (80)**	**Dorval et al. (59)**
***HLA-DQA1***					
rs1129740	X	X^*^	X^*^	–	X
rs1071630	X	X^*^	X^*^	–	X
rs9272729	–	–	–	X	–
***HLA-DQB1***					
rs4642516	–	X	–	–	–
rs3134996	–	X	–	–	–
rs1063348	–	–	X	–	–
***HLA-DRB1***					
rs28366266	–	–	X	–	–
***HLA*** **Alleles**	**Adeyemo et al**. (26)	**Jia et al**. (28)	**Debiec et al**. (27)	**Sekula et al**. (80)	–
HLA-DQA1*0101	X	–	–	–	–
HLA-DQA1*0201	X	–	X	–	–
HLA-DQA1*0501	–	–	–	X	–
HLA-DQB1*0201	X	–	–	X	–
HLA-DQB1*0302	–	X	–	–	–
HLA-DQB1*0602	–	X	–	–	–
HLA-DQB1*0604	–	X	–	–	–
HLA-DRB1*0202	–	X	–	–	–
HLA-DRB1*0301	–	–	–	X	–
HLA-DRB1*0701	X	–	X	–	–
HLA-DRB1*0802	–	X	–	–	–
HLA-DRB1*1302		X	–	–	–

*X, significant association*;

* *SNP in LD*.

Additional support for the involvement of the *HLA-DQ* region in SSNS came from a study of South Indian patients with NS that reported an association between HLA- DQB1^*^02 and SSNS (23). This was further validated in recent publication identifying the association of the *HLA-DR/DQ* loci with SSNS in the Japanese population through an unbiased GWAS analysis in two independent cohorts (28). In addition to confirming a previously identified HLA-DRB1^*^08:02 variant as a SSNS risk allele in this population, the study also identified DQB1^*^03:02 as a risk allele, with the presences of both alleles providing the most significant risk. However, unlike the *HLA-DQA1* alleles that appear to be risk factors in all ethnicities studied to date, the HLA-DRB1^*^08:02 and DQB1^*^03:02 variants seems to be specific for the Japanese population.

An extensive examination of SSNS associated risk alleles in three independent cohorts of NS patients was recently reported by Debiec et al. (27). This group used trans-ethnic GWAS, followed by meta-analysis and conditional analysis to interrogate a French discovery cohort of 273 children (132 Caucasian, 56 African, and 85 Maghrebian) with SSNS and an independent replication in a cohort of over 100 European (Caucasian) children. In addition to identifying significant associations with the *HLA-DQA1* region in the initial GWAS analysis of these cohorts, meta-analysis and conditional analysis revealed two variants in the *HLA-DR/DQ* region and one variant in the 3′ untranslated region of *BTNL2-HCG23-LOC101929163* that were significant across ethnicities. Burden analysis of the two *HLA-DR/DQ* risk alleles in the multi-ethnic Nephrotic Syndrome Study Network (NEPTUNE) cohort demonstrated an increase in SSNS risk, a decrease in age at onset of disease, and an increase in chances of complete remission associated with patients bearing multiple risk alleles. One of the *HLA* risk alleles (rs28366266) lies upstream of *HLA-DBR1*, while the other (rs1063348) is a SNP in the 3′ untranslated region of *HLA-DQB1* that is reported to reduce *HLA-DRB1, HLA-DRB5*, and *HLA-DQB1* gene expression in the NEPTUNE cohort. This trans-ethnic analysis suggests that like *HLA-DQA1*, variants in *HLA-DQB* and *HLA-DR* regions may also be universal risk factors for SSNS. Furthermore, they demonstrate that these risk alleles may also affect the expression of other genes in the *HLA* regions, providing a complex pattern of disease inheritance that will require further studies.

#### Risk Variants Outside of the MHC II Locus

The replication of associations between *HLA-DR/DQ* alleles and SSNS in multiple independent cohorts regardless of ethnicity suggests that this region plays a critical role in SSNS pathogenesis. However, these variants fail to explain the phenotypic and ethnic variation observed in SSNS patients, or the lack of renal involvement in the numerous immunologic disorders associated with *HLA* variants. This suggests the presence of additional genetic factors outside of the *HLA* region that increase the susceptibility to renal disease. This “trigger, bullet, and target” idea proposes that much like the connection between *HLA* and *PLA2R1* gene variants in adult membranous nephropathy, *HLA* variants in SSNS act as the trigger for unknown factors that connect immunologic dysfunction to renal disease (79). While the lack of strong SSNS genetic associations outside of *HLA* regions indicate these unknown factors may be diverse even within ethnicities, recent reports have identified rare variants in two SSNS candidate genes outside of the classical *HLA* region, *PLCG2*, and *BTNL2* that may contribute to disease (22, 27).

In the same cohort of South Asian NS patients used to identify associations between classical *HLA-DQA1* variants and SSNS, Gbadegesin et al. performed rare variant gene set–based analysis to identify other candidate risk loci. The best signal from this analysis was found in *PLCG2* (*P* = 7.8 × 10^−5^). Phospholipase C gamma-2 (*PLCG2*) encodes a critical signaling enzyme that is required for the production of inositol triphosphate (IP3) and diacylglycerol (DAG) in response to growth and immune system receptor activation. Mutations in *PLCG2* affect lymphocyte signaling and differentiation with hypermorphic variants associated with autoimmunity disorders in humans and glomerulonephritis in mice (81–83). A recent report has identified compound missense variants in *PLCG2* as a cause of disease in a set of twins with SSNS (84). They demonstrate that these rare variants are hypermorphic, resulting in an increase in B cell calcium flux, which is associated with an increased immune response. While the contributions of *PLCG2* to adaptive immunity have been extensively studied, the effect of these variants on GFB integrity have not been examined, despite its robust expression in the glomerulus (85). With a known role in intracellular calcium regulation, it is plausible that *PLCG2* variants may directly contribute to podocyte injury, especially with the discovery of variants in a related PLC enzyme, *PLCE1*, as a cause of NS with variable therapy response (Figure 1) (66). Recently, expression quantitative loci analysis of NS patients in the Nephrotic Syndrome Study Network (NEPTUNE) identified *PLCG2* as one of the most highly regulated genes in the glomerulus, suggesting a possible role for *PLCG2* modulation in NS (85). Further studies will be required to determine if variants in *PLCG2* are truly capable of acting as a monogenic cause of disease or if it serves as a contributing genetic factor that may or may not be specific for some ethnicities.

In the GWAS analysis of SSNS patients recently reported by Debiec et al., a missense variant in exon 3 of butyrophilin like-2 (*BTNL2*) was found to be the lead SNP in the African patients in their French discovery cohort. Subsequent transethnic meta-analysis in all four SSNS cohorts with conditioning for two *HLA* loci SNPs (rs1063348 and rs28366266) revealed a significant SNP located in close proximity to the 3′ UTR of *BTNL2*, providing additional support for a potential role in SSNS pathogenesis. *BTNL2* encodes a MHC Class II associated transmembrane protein that is involved in immune response regulation. Like *PLCG2*, variants in *BTNL2* are associated with several disorders involving elevated auto-immune responses due to its role as a negative regulator of T cell proliferation and cytokine release (79, 86, 87). The precise connections between *BTNL2* variants and the loss of GFB integrity is unclear but the increased release of inflammatory cytokines due to a loss of BTNL2 activity may serve as an additional immunologic insult capable of disrupting GFB integrity in the presence of additional podocyte related gene defects (Figure 1).

#### Candidate Genes

There are also several reports of genetic polymorphisms in genes such as *CYP3A4, CYP3A5, ABCB1*, and *GR* that can affect response to immunosuppressants in children with NS (88–94). Clearly, this additional variable will complicate treatment strategies for NS and could account for some of the variable response to immunosuppression in patients with both SSNS and SRNS. Aside from the obvious connection between glucocorticoid receptor (GR) function and corticosteroid response, the products of *CYP3A4, CYP3A5*, and *ABCB1* are required for metabolism of calcineurin inhibitors like tacrolimus, providing biologic relevance to these variant associations. However, while associations have been identified between variants in *GR* and *ABCB1* and steroid response in patients with NS, none of these genes have been found to be associated with increased incidence of SSNS (Table 4) (95–103). Additional associations between biologically relevant molecules and steroid response or relapse rates in NS patients including variants in genes encoding important signaling molecules such as Tumor necrosis factor alpha (*TNF*α), Angiotensin-converting enzyme (*ACE*), Interleukin 4 (*IL4*), and Interleukin 12 (*IL12B* promoter) have been reported (Table 4) (104–108). However, unlike the studies of *HLA* gene associations, these SSNS candidate gene studies have often lacked unbiased genome or exome wide analysis and most of the findings have not been replicated by other investigators in either gene/variant specific or unbiased genome wide studies. While it is plausible that SNPs in biologically related molecules can affect patients responses to immunosuppression, further large scale unbiased studies will be required to determine the role of these candidate genes/variants in the pathogenesis of SSNS. The candidate variants/genes identified to date in studies of childhood SSNS and their associations with disease are listed in Table 4.

**Table 4:** Candidate gene polymorphism studies in childhood SSNS.

**Gene**	**Association**	**Total NS cohort (SSNS)**	**Ethnicity**	**References**
*ABCB1 (MDR1)*	Steroid response	120 (80)	Egyptian	(95)
	Steroid response	170 (69)	Korean	(96)
	Steroid response	150 (50)	Egyptian	(97)
	NS/Steroid response	63	Tunisian	(98)
	Steroid response	74 (58)	Chinese	(99)
	Steroid response	216 (137)	North Indian	(100)
	Steroid response	138 (92)	Egyptian	(101)
	Age/Steroid response	46 (33)	Slovakian	(102)
*GR (NR3C1)*	Steroid response	154	Chinese	(103)
*TNFα*	Steroid response	150 (100)	Egyptian	(104)
*IL-4*	Frequent relapse	55 (55)	Chinese	(105)
*ACE*	NS/Frequent relapse	227 (144)	Turkish	(106)
	Steroid response	125 (90)	Indian	(107)
*IL12B*	Steroid dependence	79 (79)	German	(108)

## Future Directions

**Figure 2:**
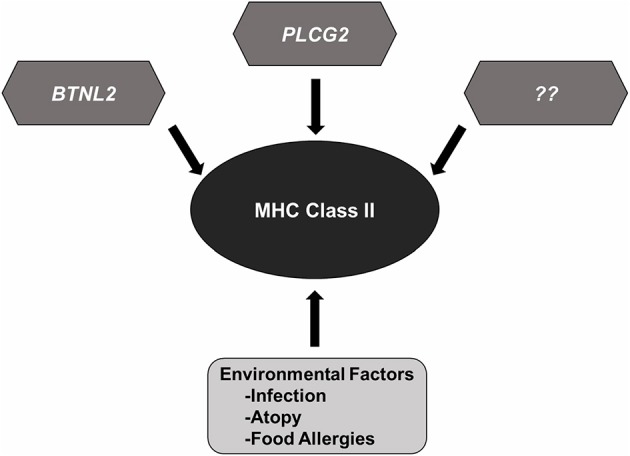
MHC Class II variants in SSNS. Accumulated genetic evidence has identified MHC Class II variants as a central component to SSNS pathogenesis. Additional risk variants such as those reported in *BTNL2* and *PLCG2*, and other rare risk variants (race or none race specific) are likely to be involved as well. Environmental factors such as infection, atopy, and food allergies likely contribute to disease through increased activation of immune system.

Despite significant advances in our knowledge of the genetic mechanisms underlying the pathogenesis of NS, several gray areas still remain. The genetic diversity of MHCII loci in different populations suggests a central role for adaptive immunity in the development of SSNS and immune-mediated diseases. Variants in the *HLA-DQA1, HLA-DQB1*, and *HLA-DRB1* appear to be risk factors for SSNS regardless of ethnicity. This universal significance and the high prevalence of variants in these genes despite low incidence of SSNS in the general population suggests that most MHCII variants require the presence of additional genetic and non-genetic factors to drive the development of SSNS, and possibly explain the variability in disease prevalence and severity between different ethnicities (Figure 2). Some of these factors include environmental influences such as infections, atopy, and food allergies that can cause dysregulation of the immune system. However, the precise connections between immunologic dysregulation and podocyte dysfunction remain unclear. Understanding how rare genetic variants such as those in *PLCG2* and *BTNL2*, act in concert with the more common MHCII variants to predispose NS patients to disease most likely holds the key to a better understanding of disease pathogenesis and identification of specific and non-toxic therapeutic targets. Such studies will require large patient cohort and therefore robust international collaborations.

Within SSNS, it is possible that there are at least two distinct groups based on disease mechanisms. The first group will be patients with complex disease mechanisms due to interplay of environmental factors and disease risk variants in the MHC II and non-MHC loci. This group most likely represents the majority of children with SSNS. On the other hand it is possible that there is another very small group with disease driven primarily by dysregulation of pathways that are responsible for maintaining normal function and structure of the podocyte. Most of the children with monogenic SSNS will probably belong to this latter group.

**Figure 3:**
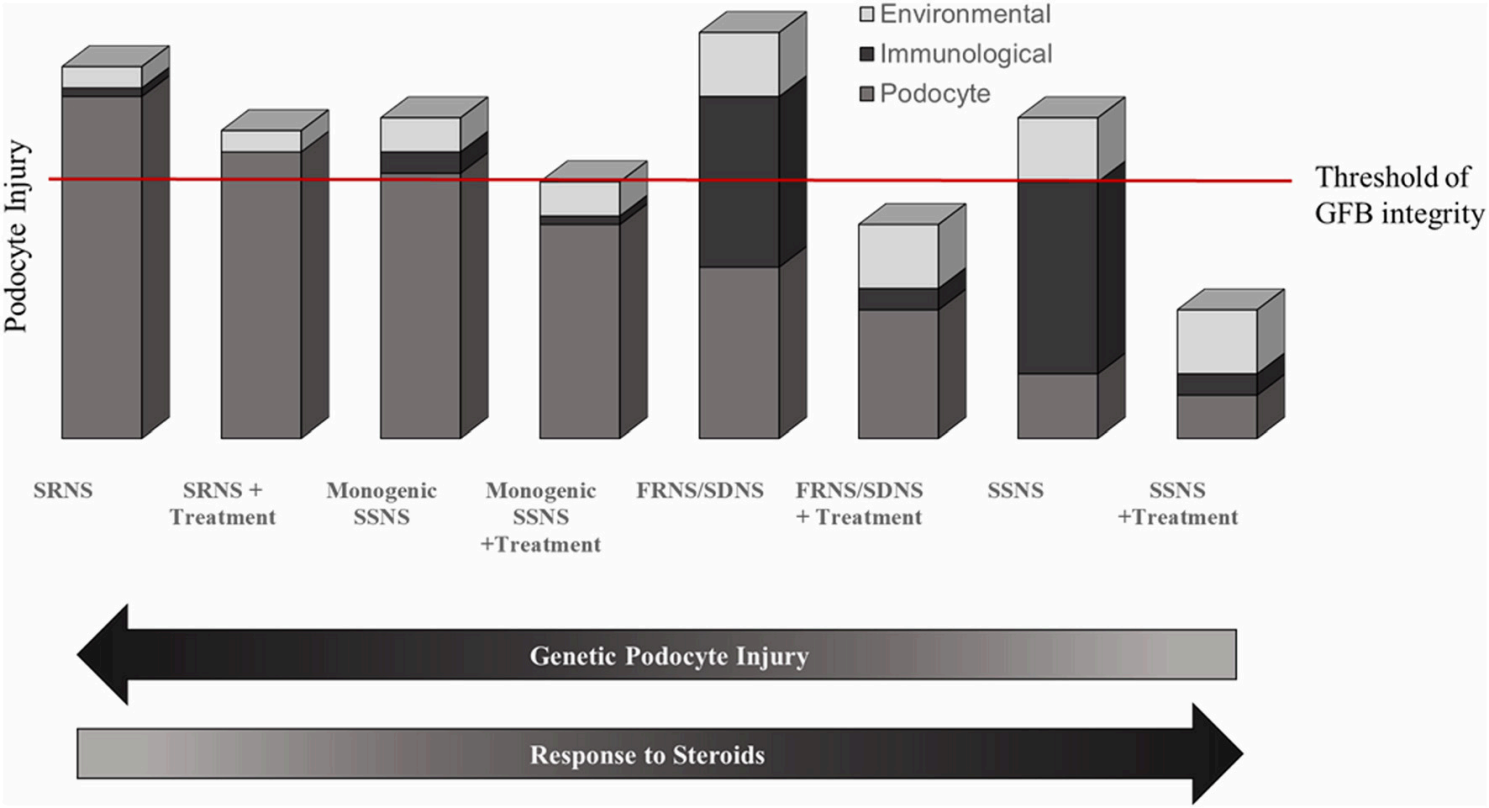
Genetic and environmental contributions to NS. Evidence suggests that SSNS results from combination of immunological and environmental insults to podocyte in a genetic background that is susceptible to podocyte injury and that there is likely a negative correlation between therapy response in NS patients and the degree of genetic podocyte injury. In a simplified view of Nephrotic Syndrome, there is likely a threshold of podocyte injury at which glomerular filtration barrier integrity is lost (red line). In SRNS, the loss of GFB integrity results primarily from genetic insults to podocyte viability that is too profound to overcome with any beneficial cytoskeletal effects of immunosuppressants. Monogenic cases of SSNS likely have less genetic podocyte injury than SRNS, such that immunosuppressive therapy is largely successful at restoring GFB integrity but the overall podocyte viability is still damaged enough to allow environmental factors to influence remission status. More common and polygenic SSNS have a larger contribution of immunological insults to podocyte, which results in a more robust response to immunomodulatory therapy. Frequent relapsers likely have a higher level of baseline podocyte injury than infrequent relapsers.

While SSNS and SRNS are generally considered to be distinct entities with diverging pathologies, recent evidence suggest that these conditions exist as endpoints on a spectrum of NS disease. The response to therapy appears to be determined primarily by the degree to which podocyte viability is directly affected by deleterious genetic variations (Figure 3). In a highly simplified view, it is likely that there is a threshold of podocyte injury needed for loss of GFB integrity and development of NS. This podocyte injury may be a direct result of genetic variants or may occur as an accumulated effect of genetic, environmental, and immunological insults. The majority of variants associated with SRNS cause direct podocyte injury that is too far above the threshold of GFB integrity to allow for disease remission following use of immune modulating therapies (Figure 3). Monogenic SSNS and partially steroid responsive NS variants on the other hand, likely result from variants that directly affect podocyte health to a lesser degree than most SRNS associated variants. Disease phenotypes in these patients can be partially ameliorated by the beneficial podocyte cytoskeletal effects of immunomodulatory medications (Figure 3). The more common and complex forms of SSNS are likely to be driven primarily by variants in components of the immune system such as *HLA* genes with large effects and contributions from variants in genes outside of the HLA region that may be race specific (109). Many of these contributing variants likely affect podocyte viability, leaving the patients susceptible to immunologic and environmental insults to podocyte health. Individuals with frequently relapsing or steroid dependent NS likely have higher cumulative podocyte injury levels than those with SSNS who are non-frequent relapsers (Figure 3). This high baseline level of injury allows for rapid progression from remission to relapse. The development of resistance to immunotherapy in some SSNS patients is likely the result of the continued environmental and immunological insults to podocyte health driving injury beyond a point where normal GFB function can be restored.

If this general hypothesis is correct and cumulative podocyte injury is the driving force behind all forms of NS regardless of therapy response, then the key to treating nephrotic syndrome more effectively lies in identifying ways to restore podocyte viability. While targeting podocyte injury in SRNS patients may appear obvious, this strategy should be extended to SSNS patients as well. By focusing on podocyte health, we may be able to reduce the chances of relapse or steroid dependency in SSNS patients and avoid the harmful side-effects of immunosuppression, even if only by lowering the effective dose. This strategy will rely on our ability (1) to determine the precise mechanisms by which known immunological variants affect podocyte viability, (2) to identify novel variants that contribute to NS pathology through continued genetic analysis of large patient cohorts, and (3) to determine the key signaling pathways in podocytes onto which multiple pathogenic variants converge. Identification of these common molecular pathways is a critical step toward identification of novel effective therapeutic targets. A possible preliminary approach in this effort would be to examine changes in gene transcription during relapse and remission of SSNS. Through the knowledge acquired from studies such as these, we can start to identify potential druggable therapeutic targets.

## Conclusion

In conclusion, evidence from published studies suggest that the vast majority of SSNS cases are likely due to a complex interaction between variations in adaptive immunity, factors innate to the kidney, environmental factors and other population-specific rare genetic variants. A first step to understanding this complexity will be identification of rare genetic variants that are acting in concert with adaptive immunity to predispose patients to SSNS. Such a study can only be accomplished by large scale international collaborative studies. In addition, integration of data from such studies with epidemiologic studies and other omics study will ultimately lead to better understanding of disease mechanisms and identification of more effective and non-toxic therapies for SSNS.

## Author Contributions

All authors contributed to the writing of this manuscript. BL created figures and tables. BL and RG edited the manuscript.

### Conflict of Interest Statement

The authors declare that the research was conducted in the absence of any commercial or financial relationships that could be construed as a potential conflict of interest.
